# Pediatric Sports: The Mental Health and Psychological Impact of Sport and Injury

**DOI:** 10.3390/jcm14124321

**Published:** 2025-06-17

**Authors:** Elaine Xu, Dylan N. Greif, Patrick Castle, Sarah Lander

**Affiliations:** 1 School of Medicine and Dentistry, University of Rochester, Rochester, NY 14642, USA; 2 University of Rochester Medical Center, Rochester, NY 14642, USA; dylan_greif@urmc.rochester.edu (D.N.G.); patrick_castle1@urmc.rochester.edu (P.C.)

**Keywords:** pediatric sports, mental health, pediatric injury, sport psychology

## Abstract

Youth sport participation provides undeniable physical, emotional, and social benefits. However, the current landscape of pediatric athletics has shifted toward early sports specialization (ESS), year-round training, and heightened competitive pressures. This has led to an increased prevalence of overuse-related traumatic injuries in adolescent patients, as well as increased risk of worsening mental health due to burnout, depression, suicide, and general psychological distress. There are numerous innovations and solutions aimed at addressing the increased risk of injury associated with current sporting trends, such as neuromuscular training programs, delayed specialization, promotion of free play, and pediatric specific surgical techniques mindful of future growth, such as those seen for anterior cruciate ligament reconstruction (ACL-R). However, the social factors associated with an injury remain problematic and are not adequately addressed; these include social isolation, depression, anxiety, and academic decline. Sport psychology is a promising solution to address many risk factors associated with poor performance, address the challenges associated with injury, and increase return-to-play in adolescent sports medicine. Integrating sport psychology into pediatric sports medicine offers the ability to directly address the emotional and cognitive demands of injury and recovery. Emphasizing mental health support and redefining success in youth sports—prioritizing enjoyment, personal growth, and long-term health over scholarships and professional aspirations—are key steps in preserving the overall benefits of pediatric sport participation. Yet sport psychology remains often underutilized and has been slow to gain traction, particularly in youth sports. This editorial serves to highlight the current state of mental health advocacy in pediatric sports medicine and how sport psychology can help young athletes manage the mental stress of high-performance athletics and mitigate the detrimental effect of injury and delayed return to sport.

## 1. Introduction

In 2020, over one-half of children between the ages of 6 and 17 years participated in youth sport [1]. Youth sport participation is associated with various benefits in various domains of pediatric development, ranging from physical to social and psychological in nature. It is well documented that organized youth sport is associated with improved cardiovascular fitness levels, social and life skill development, improved emotional regulation and ability to cope with stress, and decreased risk of mental health problems such as depression and anxiety [2]. However, despite the benefits of sport participation, there are some worrying trends, namely, early sport specialization in younger athletes and increased pressure to compete with higher intensity year-round [3]. The motivations behind sport specialization are multifactorial and include the athlete’s or parent’s desire for success, need for collegiate scholarships, and professional aspirations.

Regardless of the motivation behind these trends, today’s athletes, particularly those encouraged to specialize early on, are at risk of social isolation from peers, emotional dysregulation, burnout, and overuse injuries [3]. In addition, injuries associated with sport result in time away from sport, school, events, and friends, compounding a sense of isolation, lack of confidence in return-to-play, and potential loss of sense of identity [4]. To counter this, sport psychology, which has existed to help optimize outcomes in healthy athletes for centuries [5], has now begun to address the rising prevalence of mental issues plaguing modern day athletes [6,7]. However, while there is abundant literature demonstrating that sport psychology can improve performance and address mental health, there is limited literature addressing the role of sport psychology in helping athletes cope after injury. In this editorial we explore the detrimental effects injury may have on an athlete’s psyche, the mental pressures affecting athletes today, what role sport psychology may play in optimizing return to sport, barriers to use, and future directions. It is our hope this editorial serves as an important call to action to make the concept of sport psychology more readily available to our athletes.

## 2. The Current Mental Health Issues Plaguing Athletes

In 2023, one out of five children between the ages of 12 and 17 had a mental or behavioral health diagnosis, with adolescent athletes also included in that statistic [8]. Mental health continues to be a crisis, particularly amongst our youth, with anxiety and depression being the most diagnosed disorders. There is inconclusive evidence on whether youth athletes suffer from mental health issues more or less than their peers, as in theory, while sport participation has historically lowered the risk of acquiring a mental health diagnosis [9], rates of anxiety and depression in elite athletes are now over 30%, a trend in the wrong direction [10].

In addition to the common stressors’ athletes in particular face within their sport (i.e., overtraining, burnout, ability to perform at a high level professionally or for academic scholarships, etc.), clinicians must not forget that our young athletes are also subjected to the same “exposome” as their peers. Vodovotz et al. describe the exposome as the sum of multiple harmful environmental exposures, which can range from more global examples such as the global COVID-19 pandemic to more community-based or individual exposures such as food insecurity, social inequality, or lack of proper education [11]. We must also recognize that over the last decade, the onset of social media usage has led to a profound cultural change in how adolescent athletes interact with their peers and the world. In a recent 2023 Gallup Familial and Adolescent Health Survey, teenagers were reported to spend an average of 4.8 h per day on social media platforms [12]. Social media usage among youths has been linked to chronic sleep deprivation, mental distress, and emotional dysregulation [13]. A recent study by Fiedler et al. demonstrated that the influence of social media on youth athletes in particular may vary by performance level, as lower-performing athletes had lower mental health scores compared to elite youth athletes that used social media [14].

Overall, athletes appear to be suffering under increased mental strain now more than ever, and action must be taken to address mental health in athletes, especially at younger ages.

## 3. The Psychological Impact of Musculoskeletal Injuries

While musculoskeletal (MSK) injuries are often approached primarily from a physical recovery perspective, their psychological impact can be profound and long-lasting, especially for young athletes. Recovery encompasses not just physical healing but also emotional and cognitive adjustments, which can significantly affect an athlete’s overall well-being and performance.

As stated previously, the emotional toll of MSK injuries is driven by several factors, including social isolation, loss of athletic identity, fear of the future, and financial stress. These psychological states can delay physical recovery and have a lasting impact on long-term mental health. A systematic review by Marconcin et al. found that MSK injuries increase the risk of depression among athletes; however, the review also concluded that this relationship is bidirectional, with depression increasing the risk of subsequent injuries [15]. This suggests that athletes experiencing depressive symptoms following an initial injury may be at heightened risk of re-injury. Beyond depression, studies have shown an association between MSK injuries and symptoms of post-traumatic stress disorder (PTSD). A study by Padaki et al. on youth athletes after ACL rupture found that 87.5% of participants experienced avoidance symptoms, 83.3% had intrusion symptoms, and 75% exhibited hyperarousal symptoms, indicating significant psychological trauma in these young athletes [16].

One of the most prevalent psychological consequences of MSK injuries is fear of reinjury, or kinesiophobia. Paterno et al. observed that athletes reporting higher levels of kinesiophobia after ACL rupture had increased rates of re-injury [17]. These athletes also demonstrated lower activity levels and worse functional performance in the injured leg, likely due to fear that the injury had not fully healed and concerns about further damage. This fear can hinder rehabilitation efforts, delay or prevent return to sport, and potentially increase the likelihood of re-injury. In fact, the study found that athletes with high levels of fear were 13 times more likely to suffer a second ACL tear within 24 months of returning to sport. Systematic reviews, such as that of Nedder et al., confirm how overcoming fear of re-injury and reducing kinesiophobia is critical for athletes to resume normal activities and perform at pre-injury levels [18].

Specifically, in young athletes involved in early sport specialization, the mental health burden of injury is particularly pronounced. For many, sense of identity is closely tied to their role as competitive athletes. When an injury requires time away from sport, it can lead to the loss of this identity, manifesting as feelings of purposelessness or diminished self-worth. The strength of athletic identity is greater among youth athletes with higher levels of sport specialization and those who began their primary sport at an earlier age, making them more vulnerable to mental health challenges when their identity is disrupted by injury [19]. In a systematic review, Park et al. found that pediatric athletes with strong athletic identity linked to a particular sport were especially vulnerable to the development of depressive symptoms after MSK injury [20]. These findings can be compounded by competition for college scholarships or performance on a professional level. To protect young athletes, it is crucial to acknowledge that loss of athletic identity can have as much of an impact on recovery and return to sport as the physical injury itself.

Overall, though physical recovery is essential for determining when an athlete is ready to return to sport, these findings emphasize the importance of psychological readiness as well. Ardern et al. in particular further illustrated this when they determined that psychological readiness was the strongest predictor of return to pre-injury level of activity [21]. For every one-point increase in psychological readiness, as measured by the ACL-Return to Sport after Injury (ACL-RSI) scale, the odds of returning to pre-injury activity levels doubled. When Senorski et al. interviewed male athletes after both their initial ACL tear and subsequent ACL re-rupture, athletes frequently regretted not talking to a sport psychologist before being cleared for return to sport and not having such a resource to process the mental trauma that comes with a long rehabilitation or potentially having to pivot away from athletics entirely [22]. This highlights the need for rehabilitation strategies that address both physical and mental health.

Unfortunately, many current strategies, such as neuromuscular training, delayed specialization, promotion of free play, and pediatric-specific surgical techniques, focus primarily on physical improvements, without adequately addressing mental health concerns. This represents both a gap in current care but also an opportunity to implement sport psychology into post-injury recovery.

## 4. Understanding the Role of Sport Psychology

Sport psychology has the potential to help athletes manage the mental health challenges that arise from MSK injuries. By addressing both the emotional and cognitive aspects of recovery, sport psychologists can enhance mental and physical outcomes for injured athletes. Research has shown that athletes often rely on poor coping strategies during recovery. One study found that athletes with poorer return-to-sport outcomes used ineffective coping mechanisms such as unrealistic wishful thinking, emotional venting, denial, and disengagement [23]. This illustrates the need for psychological intervention to replace these harmful behaviors with more effective coping strategies. Several psychosocial interventions have been established to improve athletic performance by targeting the athlete’s mindset, including goal setting, mental practice/imagery, self-talk, exposure therapy, and relaxation techniques.

Goal setting is primarily used as a motivational tool to drive effort toward achieving an outcome. It offers cognitive benefits, such as enhancing focus, directing attention, increasing persistence, and fostering problem-solving strategies. Though most studies, such as that of Kyllo et al., have shown that goal setting can improve general athletic performance [24], Evans et al. specifically found that goal setting in the context of injury increased self-reported adherence to rehabilitation programs [25]. While they did not comment on return to sport or pre-injury level, their findings indicate that goal setting and self-motivation can directly influence an athlete’s commitment to the recovery process.

Mental practice, or imagery, involves the mental rehearsal of motor movements without physical execution. Athletes can apply this process to cognitively rehearse specific movements within their sport. This technique has been shown to improve sport performance and is widely adopted in disciplines such as gymnastics, tennis, and figure skating [26,27,28,29,30,31]. Many athletes, particularly those competing at higher levels, report using mental imagery, but often without structured sessions, highlighting an opportunity for further development in this area [31]. In the context of injury rehabilitation specifically, four distinct types of mental imagery techniques have been described in the literature, expanding beyond just the rehearsal of motor skills [29,32,33]. Healing process imagery involves athletes visualizing the healing of the injured body part, though this technique may be limited by the athlete’s understanding of the injury’s anatomy, which can restrict participation. Pain management imagery focuses on teaching athletes techniques to cope with pain associated with injury. Rehabilitation imagery encourages athletes to visualize overcoming challenges throughout the rehabilitation process. Finally, performance imagery involves mentally rehearsing physical skills related to their sport. While the effectiveness of these techniques varies depending on the athlete’s ability to use imagery, there is evidence that athletes can improve their imagery skills with practice [34]. Preliminary research suggests that motor imagery can improve functional outcomes following ACL reconstruction, with athletes demonstrating greater knee strength and reduced anxiety about re-injury compared to controls [35]. Another study, investigating mental imagery following a grade II ankle sprain, found that athletes participating in imagery had improved muscular endurance compared to those who did not [36]. While these techniques show promise, the existing literature remains limited, primarily involving adults and small sample sizes. Additionally, the impact of mental imagery on return to sport or achieving pre-injury performance levels has not yet been fully explored.

Self-talk refers to the internal dialogue athletes use during training and competition, influencing their mindset, emotional state, focus, and confidence. Positive self-talk helps athletes stay motivated, overcome setbacks, and manage stress, while negative self-talk can impede performance. Athletes that rely significantly on external motivation require psychological support to increase intrinsic motivation and self-efficacy [37]. In the context of injury recovery, self-talk can be a powerful tool for building resilience and maintaining a positive outlook. Hatzigeorgiadis et al. found that self-talk has a moderate positive effect size on sport performance [38], though not much literature exists on this technique in the context of recovering from injury.

In vivo exposure therapy gradually exposes athletes to functional tasks that elicit fear, with the goal of reframing maladaptive perceptions of these tasks. Originally developed for patients with chronic low back pain, research has shown that this therapy effectively reduces fear of movement and re-injury while increasing physical activity levels [39,40]. Although it has not been extensively studied in athletes, there is significant potential for this technique, particularly for managing pain-related fear following MSK injury.

Relaxation techniques are commonly employed to reduce stress, anxiety, and both mental and physical strain in injured athletes. These techniques help athletes become more aware of their physiological and psychological states, enabling them to regulate their arousal levels for optimal performance. One widely used relaxation method is deep breathing, which helps athletes calm down and refocus on the present moment. In two studies exploring the benefits of deep breathing exercises, athletes reported improved mood, reduced anxiety, and greater readiness for competition [35,41]. Progressive muscle relaxation (PMR), in which athletes tighten and then relax specific muscle groups to achieve total relaxation over multiple sessions, is also another potent relaxation tool. Battaglini et al. found that PMR reduced anxiety and stress among adolescent basketball players [42], while Hashim et al. showed that PMR reduced depression and tension in adolescent soccer players [43]. If junior athletes can adopt stress-reducing techniques early on in their careers, they can subsequently develop proficient coping mechanisms for dealing with stress, especially after injury, which can thus be viewed as a challenge on the path to success rather than a barrier [44]. However, there is sparse literature demonstrating the effectiveness of these tools during injury recovery with a sport psychologist.

These techniques highlight the potential impact of psychological interventions among athletes. Many of these strategies, such as goal setting, mental practice, and self-talk, can be implemented independently by athletes once they have a foundational understanding introduced by a sport psychologist or a certified mental performance consultant, a specialist trained to help athletes develop mental skills to enhance their performance. With simple, actionable behaviors like setting measurable goals or incorporating deep breathing exercises, athletes can integrate these techniques into their daily routines, enhancing their rehabilitation process without requiring substantial time or constant supervision. However, more research is needed, as current studies on the role of sport psychology specifically following MSK injuries are limited, and very few have explored these techniques in pediatric or adolescent populations recovering from such injuries.

## 5. Evidence of Success

Because of the increased urgency in adopting mental health into athletics, there is evidence to suggest that post-operative psychosocial intervention is beneficial for athletes. Naderi et al.’s meta-analysis demonstrates that psychosocial interventions such as guided imagery, positive reinforcement, muscle relaxation, and in vivo exposure therapy in conjunction with standard rehabilitation protocols can improve kinesiophobia after ACL-R, though their conclusions are based on a small sample size [45]. One study within Naderi et al.’s review reported reduced fear of re-injury and improved kinesiophobia after ACL-R in a professional athlete population when a psychologist assisted in post-operative muscle relaxation exercises, though the follow-up and sample size were limited, and a specific sport psychologist was not employed [46]. Other systematic reviews have also concluded that relaxation/guided imagery, positive self-talk, goal setting, etc., can facilitate positive mood changes, pain management, and rehabilitation adherence, but no study has directly assessed the role of psychosocial interventions with a trained sport psychologist on post-operative functional outcomes and return to sport [6].

Because of the above, there is a study that demonstrates inconsistent findings as to the actual benefits of post-operative psychosocial interventions [47]. Coronado et al. found that the limited patient sample sizes and large variations of methodology assessing patient outcomes made it difficult to recommend psychosocial interventions for improving functional recovery and return to sport, and though Naderi et al. found studies that saw improvements in post-operative kinesiophobia, they could not comment on whether functional outcomes or return to sport improved with any sort of psychosocial intervention. There also appears to be a lack of literature addressing whether a trained sport psychologist versus an athletic trainer, physical therapist, or coach driving the psychosocial recovery progress improves post-operative outcomes [48], despite the call to employ sport psychologists trained in cognitive behavioral therapy to address other aspects of sport participation such as athlete burnout, low self-confidence, and screening for depressive symptoms [5,49]. There is also a lack of studies assessing the role of sport psychology in the pre-operative phase after injury, when, for example, patients with an ACL injury have a delayed surgical date due to the need to perform pre-rehabilitation, which may serve as an important timepoint to begin any sort of psychological intervention as this treatment pathway becomes more commonplace [50].

In conclusion, there is evidence suggesting psychosocial interventions may assist in the recovery phase, particularly after ACL-R, but more work needs to be performed to assess if and how sport psychology can improve post-operative functional outcomes and return to sport, and when such interventions should be employed (before/after injury, during the recovery phase, etc.). Answers to these questions will help guide proper use of this modality for both professional and recreational athletes alike.

## 6. Barriers to Utilization

Despite calls to encourage athletes to engage in mental health services with a sport psychologist, utilization rates of these services remain varied at best [51]. Though the stigma of mental health in sports has seen a significant shift in the past two decades, the stigma of using mental health services in young athletes still exists [52,53]. The factors on an individual level that prevent use of such services in adolescent and college athletes range from internal factors of the athlete to external pressures. For example, multiple studies have shown that male athletes, particularly those with strong adherence to masculine ideas, are less likely to engage in sport psychology services [51]. Student athletes have reported an increased focus on self-reliance to push through adversity alone, as well as lack of time to seek out and use these services even when made readily available [54]. Team sports, thought to encourage socialization and reliance on others, also seem to promote negative views of sport psychology, which is only exacerbated in male-specific environments [55]. Finally, athletes often connect with sport psychologists because of their understanding of sporting identities and experience in high-level competition. However, there is a shortage of psychologists specifically trained in sports psychology who can relate to athletes’ experiences and share that same athletic identity [54].

Unfortunately, a major reason for lack of sport psychology adoption hinges on one of the most important external factors to an athlete: their coach. Multiple studies have demonstrated that a coach’s desire to maintain control over all team dynamics, or their personal negative perceptions of such services, discouraged athletes from seeking support from a psychologist [56,57,58]. Athletes reportedly conceal pain or discomfort in favor of promoting a false mentality of toughness out of fear of being perceived as weak by their coaches [59]. Such factors play a negative role on return to sport after injury and need to be addressed [60]. Therefore, the adoption of sport psychology must address the stigma that persists amongst coaching staff, particularly at the high school and college levels.

Finally, lack of access to sport psychology plays a role in its continued underutilization. Administrators for large athletic departments either are not hiring sport psychologists out of lack of belief in the service or are unaware of what type of professionals need to be hired to address such needs [51]. There is also the associated cost of hiring additional staff within an athletics department to serve this role, a cost not every school at the collegiate or high school level can bear. While the cost of sports injuries has been studied (including time-loss estimates for professional athletes), there is no cost-based analysis of time or money saved with intervention of a sport psychologist, making it difficult to discuss any financial incentives to hire sport psychologists within financially strained environments [61]. Therefore, the cost–benefit analysis of a sport psychologist must emphasize the positive individual benefits on the athlete over the financial costs of such services for wider adoption to be implemented.

## 7. Future Directions

As stated previously, though there are many physical and mental health benefits of youth sports, the mental health issues pertaining to sport participation or poor recovery, which are rising, can be devastating to younger athletes. While, as previously stated, even though adult athletes report depression, anxiety, lower self-esteem, and post-traumatic stress after an injury [62,63], the impact injury has on the mental well-being of a pediatric athlete has not been well described, nor are there studies pertaining to psychosocial intervention for pediatric athletes that have experienced a traumatic event such as a season-ending injury. In addition, there are no current studies to date that look at the long-term effects of injuries that occur in youth sports on adulthood, and there remains a distinct lack of randomized control studies assessing how seeing a sport psychologist after injury affects athletes [4].

Future directions for this topic are multifactorial. From a public health perspective, the risks of early sport specialization need to be emphasized to children and parents, as it has been shown that multisport participation does not impact athlete performance. Sports medicine specialists, pediatricians, sports psychologists, coaches, and parents need to work together to decrease the mental health stigma in youth sports. A longitudinal focus assessing psychological intervention on pediatric athletes is necessary to not only develop mental health strategies to prevent injuries, but coping mechanisms in response to injury [4]. There is a need for prospective randomized control trials assessing the impact of psychological intervention on injured athletes, and research should focus on how sport psychology can play a role with helping athletes return to sport. In addition, research should be conducted assessing how traumatic experiences during youth sports, such as injury, impacts adulthood given that there are many studies demonstrating the benefits of playing youth sports on adulthood.

Overall, though more work needs to be performed to address the mental health issues associated with competitive sport and injury, sport psychology has a role to play in addressing these gaps in care, and more research needs to be performed to assess the outcomes of such interventions. In the interim, providing immediate access to such resources if funding is available should be a priority for our athletic population, whether it be for recreational or professional participants. Developing easily adoptable programs that can be streamlined into the athlete’s everyday schedule at low cost may decrease the apprehension of incorporating such resources by coaches and their staff, both before and after an injury. Incorporating certified mental performance consultants (who already have pre-existing relationships with coaching staff due to their current work in optimizing individual athlete performance and assisting in talent development) into such programs may reduce such a stigma and thus act as an extension to sport psychologists as well.

By emphasizing the continued importance of mental health and the role sport psychologists have in the overall physical and mental growth of our athletes, in time sport psychology will hopefully be routinely adopted without the stigma associated with using such resources in years past.

## 8. Conclusions

With the importance of mental health in athletes gaining prominence in the literature, sport psychology will play a vital role in not only optimizing performance, but addressing mental health challenges associated with high-level performance, injury, and subsequent return to sport. Integrating sport psychologists and their counterparts, such as certified mental performance consultants, into athletic departments and communities alike can make the use of such resources a routine part of healthy competition and thus reduce the stigma associated with seeking help. The authors call for higher-quality evidence supporting the use of sport psychologists in addressing recovery and return-to-sport to further shed light on their important role in our sporting communities.
